# Low-Carbon UHPC Incorporating GGBS–Calcium Carbide Slag and Recycled Plastic Fibers: Mechanical Properties, Hydration, and Sustainability

**DOI:** 10.3390/ma19153277

**Published:** 2026-08-03

**Authors:** Weiliang Wang, Haoran Guo, Tianjiao Han, Qi Wang, Yanjie Wang

**Affiliations:** 1 Transportation Engineering, Beijing University of Civil Engineering and Architecture, Beijing 100044, China; 1108140023014@stu.bucea.edu.cn (W.W.); 1108140024059@stu.bucea.edu.cn (T.H.); 1108140024063@stu.bucea.edu.cn (Q.W.); 2 School of Mine Safety, University of Emergency Management, Sanhe 065201, China; 3 School of Civil Engineering, Hebei University of Engineering, Handan 056038, China; wangyanjie@hebeu.edu.cn

**Keywords:** UHPC, industrial solid waste, recycled plastic fibers, mechanical properties, hydration behavior

## Abstract

**Highlights:**

- Low-carbon UHPC was designed using industrial solid wastes (ISW) and recycled plastic fibers (RPF).
- Flexural and compressive strengths increased by up to 41.02% and 14.93%, respectively.
- Moderate ISW replacement accelerated hydration and promoted C-S-H/C-A-S-H gel formation.
- Scenario-specific selections were identified: 30% ISW-50% RPF for flexural strength and 30% ISW-30% RPF for compressive strength–flowability balance.
- The 50% ISW-50% RPF mixture reduced raw-material CO_2_ emissions by approximately 46.8% and raw-material cost by 24.71% while maintaining a compressive strength of about 150 MPa.

**Abstract:**

Ultra-high-performance concrete (UHPC) typically contains high cement and steel-fiber contents, leading to high cost and carbon emissions. This study developed a low-carbon UHPC by partially replacing cement with industrial solid waste (ISW) composed of ground granulated blast-furnace slag and calcium carbide slag, and by partially replacing steel fibers with recycled plastic fibers (RPF). The effects of ISW and RPF on flowability, mechanical properties, hydration behavior, microstructure, carbon emissions, and raw-material cost were investigated. ISW had a limited influence on flowability, whereas RPF markedly reduced flowability. Appropriate ISW and RPF contents increased flexural and compressive strengths by up to 41.02% and 14.93%, respectively. The 30% ISW-50% RPF mixture provided the highest flexural strength, while 30% ISW-30% RPF achieved the highest compressive strength with acceptable flowability. Hydration heat, XRD, SEM, and FTIR analyses showed that moderate ISW promoted early hydration and C-S-H/C-A-S-H gel formation, whereas excessive ISW caused dilution and reduced matrix compactness. Therefore, 30% ISW-30% RPF is recommended as the balanced formulation, whereas 50% ISW-50% RPF is more suitable for carbon- and cost-sensitive applications and maintains approximately 150 MPa compressive strength.

## 1. Introduction

Under the global agenda of carbon peaking and carbon neutrality, the construction materials sector is under increasing pressure to reduce its consumption of natural resources and associated carbon emissions [1,2,3]. Conventional materials such as cement and steel are associated with intensive energy use and substantial CO_2_ emissions during production, which poses significant challenges to the sustainable development of civil and mining engineering. This issue is particularly relevant to engineering applications that require both high mechanical performance and long-term durability, including infrastructure, high-rise buildings, bridges, marine structures, and underground mine reinforcement [4]. In demanding service environments, conventional concrete may struggle to simultaneously meet the requirements of strength, crack resistance, durability, and construction efficiency. Owing to its exceptional mechanical properties, dense microstructure, excellent durability, and good flowability, ultra-high-performance concrete (UHPC) has emerged as a promising material for high-performance infrastructure and specialized applications, including underground reinforcement [5,6].

However, conventional UHPC generally relies on high cementitious material content, very low water-to-binder ratios, optimized particle packing, and a large volume of steel fibers to achieve a dense microstructure and effective crack control [7]. Although these features contribute to excellent mechanical properties and durability, they also result in high environmental impacts and production costs [8]. Cement clinker production consumes considerable energy and generates substantial CO_2_ emissions, while the manufacture of steel fibers further increases energy consumption, carbon emissions, and material costs [9,10,11]. Therefore, developing eco-friendly UHPC (e-UHPC) through the partial replacement of carbon-intensive components has become an important strategy for sustainable construction materials and low-carbon mine reinforcement [12].

The incorporation of industrial solid wastes (ISW) [13,14,15] and recycled plastic fibers (RPF) [16] provides a promising approach for developing e-UHPC. Industrial by-products such as calcium carbide slag (CC) and ground granulated blast-furnace slag (GGBS) are produced in large quantities, and improper disposal may cause environmental problems such as land occupation and pollutant leaching [17,18,19]. Meanwhile, the accumulation of waste plastics has imposed long-term ecological pressure. Incorporating these waste-derived materials into UHPC can reduce the consumption of natural resources and carbon-intensive raw materials while promoting waste valorization. This approach can help achieve a balance among mechanical performance, environmental sustainability, and economic feasibility [20,21].

Alkali-activated binders derived from industrial by-products rich in reactive silica and alumina have been widely investigated as low-carbon alternatives to ordinary Portland cement. Under alkaline conditions, materials such as silica fume (SF), fly ash (FA), and metakaolin (MK) can undergo dissolution and polymerization reactions, forming cementitious products that contribute to dense microstructures [22]. Compared with traditional cement-based systems, alkali-activated binders can reduce clinker consumption, lower carbon emissions, and promote industrial waste utilization, making them a promising route for producing high-performance and low-carbon concrete [23]. Among these materials, GGBS is particularly attractive because of its high latent hydraulic activity, wide availability, and good compatibility with cementitious systems. With appropriate activation, the latent reactivity of GGBS can be effectively stimulated, producing a dense and high-strength cementitious matrix while reducing cement consumption [24].

Conventional alkali-activated UHPC generally relies on NaOH and Na_2_SiO_3_, whose high cost, handling risks, and embodied carbon may hinder large-scale application [22]. As a Ca(OH)_2_-rich industrial by-product, calcium carbide slag (CC) provides a more economical and environmentally beneficial activator for GGBS-based systems [19,25,26]. It can activate GGBS and promote the formation of calcium aluminosilicate hydrate (C-A-S-H) gel [27], thereby improving matrix compactness and strength development. This strategy follows the principle of waste-to-resource utilization and contributes to both low-carbon concrete production and industrial waste valorization [28,29,30,31]. However, research on CC-activated GGBS-based UHPC remains limited and has mainly examined hydration behavior, curing regimes, and steel-fiber-reinforced formulations [32].

Beyond binder optimization, fiber reinforcement also critically influences the mechanical performance and sustainability of UHPC. Steel fibers provide effective crack bridging and improve tensile and flexural strength but are costly and energy-intensive, whereas recycled plastic fibers (RPF) are lightweight, economical, recyclable, and corrosion-resistant [33,34,35]. Although RPF generally has lower elastic modulus and tensile strength than steel fibers, it can contribute to ductility, energy dissipation, and crack control. In underground mine environments characterized by high humidity and potential corrosion, the corrosion resistance of RPF may provide additional durability advantages. Therefore, a hybrid fiber system combining steel fibers and RPF may maintain the mechanical performance of UHPC while improving its environmental and economic sustainability [36,37,38,39]. The development of alternative fiber materials and hybrid fiber reinforcement systems has become an important research direction for e-UHPC [40,41,42]. Previous studies have shown that alkali-activated or composite binders containing slag [43], FA [44], or SF [45] can meet the strength and durability requirements of UHPC. These findings indicate that the development of e-UHPC requires the coordinated design of binder, aggregate, and fiber systems.

Although CC has recently been explored as a solid-waste-derived alkaline activator, the hydration kinetics and microstructural evolution of CC-activated GGBS-based UHPC remain insufficiently understood, particularly under steam curing. Specifically, the effects of Ca(OH)_2_-derived alkalinity on GGBS dissolution, C-A-S-H formation, and matrix densification require further clarification [46]. Moreover, the simultaneous reduction in cement and steel-fiber consumption remains underexplored [12]. The incorporation of RPF may improve waste-plastic valorization and reduce the environmental burden of UHPC, but its effects on workability, strength development, crack-bridging behavior, and fiber–matrix interaction in carbide-slag-activated GGBS-based UHPC remain unclear [47]. Consequently, the coupled effects of solid-waste-based binder substitution and hybrid steel-RPF reinforcement on hydration, engineering performance, environmental impact, and cost require systematic investigation.

To address these gaps, this study develops an e-UHPC in which a GGBS-CC blend partially replaces cement, and RPF partially replaces steel fibers on a volume basis. The effects of ISW and RPF on flowability, mechanical properties, hydration, microstructure, carbon emissions, and production cost are systematically evaluated, aiming to establish a low-carbon, cost-effective UHPC design strategy for demanding infrastructure applications, including underground mine support and repair.

## 2. Materials and Methods

### 2.1. Raw Materials

**Figure 1:**
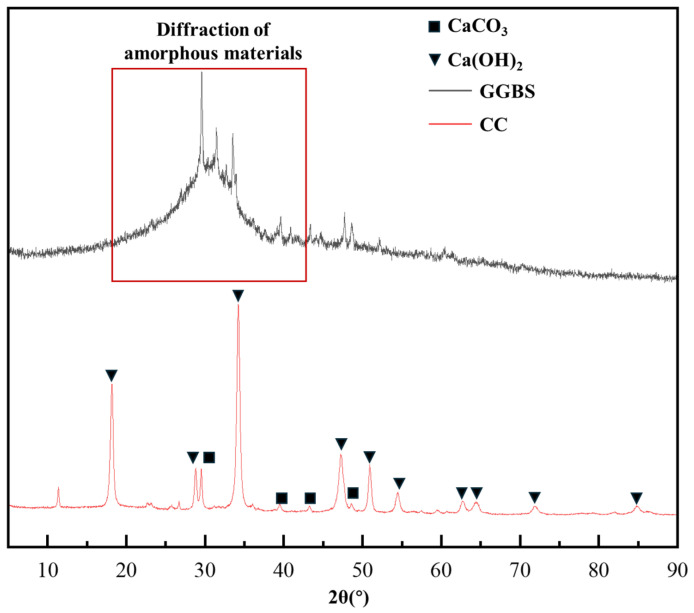
XRD pattern of GGBS and CC.

The cement used in this study was P·O 52.5 ordinary Portland cement with a specific surface area of 387 m^2^/kg, and the main performance indicators are shown in Table 1. The silica fume (SF) was a UHPC-grade product with a reactive silica content of 98% and an average particle size of 3000 mesh. The fly ash (FA) was ultra-fine sinking beads with an average particle size of 2000 mesh; its glassy spherical structure improved slurry fluidity. The ISW was composed of GGBS and CC. GGBS is a calcium aluminosilicate material rich in glassy phases and is produced by grinding water-quenched blast-furnace slag, whereas CC is an industrial waste generated during acetylene production through the reaction of calcium carbide with water. After drying and grinding, GGBS and CC were mixed at a mass ratio of 5:1. This ratio was selected with reference to previous studies [48,49] and was kept constant because the present study focused on the effect of the total ISW replacement level rather than on optimizing the activation of GGBS by varying the CC content. Cement replacement levels of 10%, 30%, and 50% by mass were selected to represent low, intermediate, and high ISW contents, respectively. The maximum replacement level was limited to 50% to ensure that cement constituted at least half of the total binder, thereby maintaining the cement-based nature of the UHPC system. This experimental design enabled the effect of the ISW content to be evaluated over a broad range while keeping the GGBS-to-CC ratio constant. SF, FA, GGBS, and CC were separately sieved through a 200-mesh sieve and then pressed into pellets at a pressure of 35 MPa. Their chemical compositions were subsequently determined using a SPECTRO XEPOS X-ray fluorescence spectrometer (SPECTRO Analytical Instruments, Kleve, Germany), and the results are presented in Table 2. Figure 1 shows the XRD patterns of slag and carbide slag. It can be observed that the slag contains a considerable amount of amorphous phase and exhibits potential alkali activity. The carbide slag consists mainly of calcium hydroxide and calcium carbonate, which provide the necessary alkalinity to promote the pozzolanic reaction.

**Figure 2:**
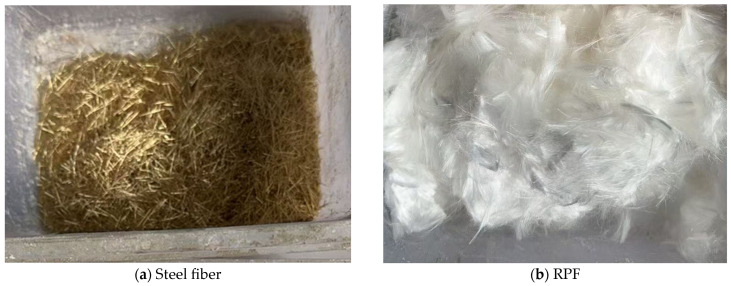
Appearance of steel fiber and RPF.

In addition, quartz sand was used as fine aggregate filling, with a density of 2.63 g/cm^3^, water absorption of 0.31% and fineness modulus of 2.42. In order to ensure fluidity, polycarboxylate superplasticizer (SP) was used to adjust the fluidity. In order to ensure the strength of UHPC, steel fibers with a diameter of 0.2 mm and a length of 13 mm were used. The tensile strength was 2760 MPa, the elastic modulus was 205 GPa, and the density was 7.85 g/cm^3^. RPF were manufactured by a recycled plastic recycling company located in Tai’an City, Shandong Province, using recycled plastic products including plastic bottles, plastic tableware and other post-consumer plastic wastes as raw materials. Waste plastics were processed into fibers through a series of procedures: cleaning, sorting, crushing, melt extrusion, drawing and cutting. The diameter of RPF is 0.05 mm, the length is 10 mm, the tensile strength is 541 MPa, the elastic modulus is 15 GPa, and the density is 0.92 g/cm^3^. The appearance of steel fiber and RPF is shown in Figure 2a,b.

### 2.2. Mixture Proportions and Specimen Preparation

Table 3 shows the mix proportions of UHPC, and the control group is UHPC without ISW and RPF. Groups G1, G2 and G3 are the e-UHPC mix ratios prepared by replacing 10%, 30% and 50% of cement with ISW. G4, G5 and G6 are the mix proportions of e-UHPC prepared by ISW and RPF. The fibers are incorporated using isovolumetric substitution. When the substitution rate of cement quality is 30%, the steel fiber is replaced by RPF in equal volume, and the substitution rates are 10%, 30% and 50%. The G7 group used RPF instead of steel fiber at a volume substitution rate of 50% based on the 50% cement substitution rate of the third group. It should be noted that the mixture design was not a full factorial design. This paper considers evaluating the effect of ISW through groups G1–G3. The effect of RPF is evaluated through groups G2, G4, G5, and G6. Additionally, group G7 is included to assess the combined effect of high-content ISW and high-content RPF.

**Figure 3:**
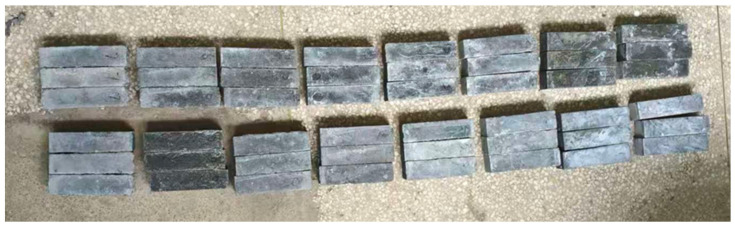
Appearance of the test specimen.

Firstly, cement, silica fume, fly ash and ISW were mixed for 2 min, and polycarboxylate superplasticizer was added to half of the water to mix well and pour into the mixed cementitious material. Mix evenly for 3 min to form a paste, then add quartz sand and the remaining water to continue stirring for 3 min. Steel fiber and RPF are added during the stirring process. Steel fiber and RPF need to be evenly spread to avoid fiber agglomeration. After the stirring is completed, it is loaded into a 40 × 40 × 160 mm^3^ mold, vibrated for 2 min, and then demoulded after standing in the room with a temperature of 20 °C ± 5 °C and a relative humidity of more than 50% for 24 h. Immediately after demoulding, it was placed in a steam curing chamber with a temperature of 90 °C + 2 °C and a relative humidity of more than 95% for 7 days for mechanical testing, as shown in Figure 3. All specimens were subjected to 90 °C + 2 °C steam curing for 7 days to ensure a consistent curing regime. The samples for the microscopic test were cut into 1 cm^3^ by the cutting machine and immersed in anhydrous ethanol to terminate the hydration. Before the test, the samples were dried in an oven at 40 °C for 24 h to remove the ethanol.

### 2.3. Testing Methods

#### 2.3.1. Flowability Test

**Figure 4:**
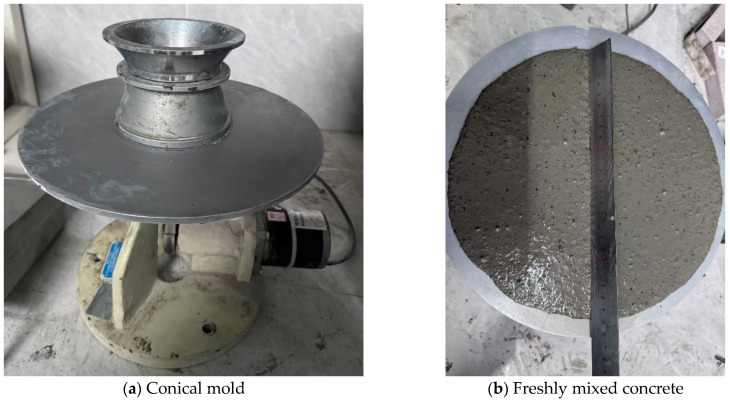
The flowability test of concrete mixtures.

Referring to the standard GB/T 2419-2005 [50] and the method for measuring the fluidity of cement mortar proposed by Zhao et al. [51], the fluidity of UHPC was measured by a jump table. First, pour the fresh concrete into a conical mold, as shown in Figure 4. Start the jump table to ensure that the mold vibrates 25 times. Then remove the mold and measure the average diameter of the mixture in the parallel and vertical directions.

#### 2.3.2. Compressive and Flexural Strength Tests

**Figure 5:**
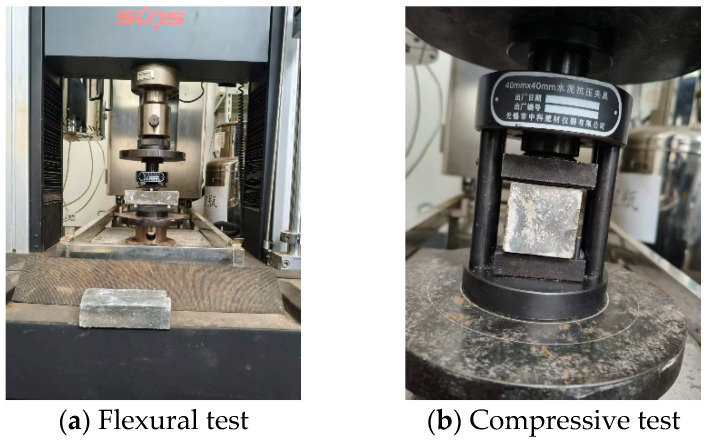
Mechanical test device.

The mechanical properties (compressive strength and flexural strength) of e-UHPC were tested. Referring to the specification GB/T 17671-2021 [52] and the experimental method adopted in previous studies [53], the flexural strength of the test block was tested at a loading speed of 0.2 mm/min, as shown in Figure 5a. After the prism specimen was broken, the cube compressive strength test mold was used to test the cube compressive strength, as shown in Figure 5b. Three flexural strength specimens were prepared for each mix ratio, and six compressive strength specimens were formed after the flexural tests were completed.

#### 2.3.3. Isothermal Calorimetry

In order to explore the effect of ISW incorporation on the early hydration reaction of e-UHPC, the hydration heat of the slurry was tested by a TAM isothermal calorimeter (TA Instruments, New Castle, DE, USA). At a constant temperature of 20 °C, 10 g of cementitious material samples were weighed according to the mix ratio, and the hydration heat within 72 h was tested after mixing with water. The results include heat flow and cumulative heat data, which are standardized based on the weight (g) of the cementitious material for each sample.

#### 2.3.4. Microstructural and Phase Characterization

(1) SEM

In order to explore the effect of ISW incorporation on the microstructure of e-UHPC, this paper uses the field emission scanning electron microscope (SEM) produced by the JEOL company (Tokyo, Japan) to analyze the microstructure of the e-UHPC test block under the acceleration voltage of 5–15 kV. The test multiples are 500 times and 3000 times, respectively.

(2) XRD

In order to obtain the phase composition of the hardened slurry, the specimens after curing were crushed, and the quartz sand was artificially separated as much as possible, and the slurry was ground to more than 200 mesh by using a mortar. For quantitative analysis, α-Al_2_O_3_ (corundum) was added as an internal standard at 15 wt% and homogenized by dry mixing in an agate mortar. The actual mass of the added corundum was accurately recorded for each sample to ensure quantitative reliability. The prepared powder was used for XRD analysis (Ultima IV from Rigaku, Osaka, Japan). The scanning range was 5–90° (2θ), the step size was 0.02°, and the scanning time per step was 0.24 s. Phase identification and full-pattern Rietveld refinement were performed using HighScore Plus 3.0. All identified crystalline phases, including quartz, calcite, portlandite, alite, belite, and corundum, were included in the refinement model.

(3) FTIR

Fourier transform infrared spectrometer (IRTracer-100, Shimadzu, Kyoto, Japan) can analyze the changes in phase composition and functional groups by absorbance, and then characterize the hydration products of e-UHPC, which can be mutually confirmed with the test results of XRD. During the test, 1 mg of sample was mixed with 100 mg of potassium bromide, and then fully ground with an agate mortar. Subsequently, the mixed samples were pressed for 1 min at 20 MPa using a tableting machine to form flakes, which were tested by infrared spectroscopy. The infrared spectrum scan range is 400–4000 cm^−1^.

## 3. Results and Discussion

### 3.1. Flowability

**Figure 6:**
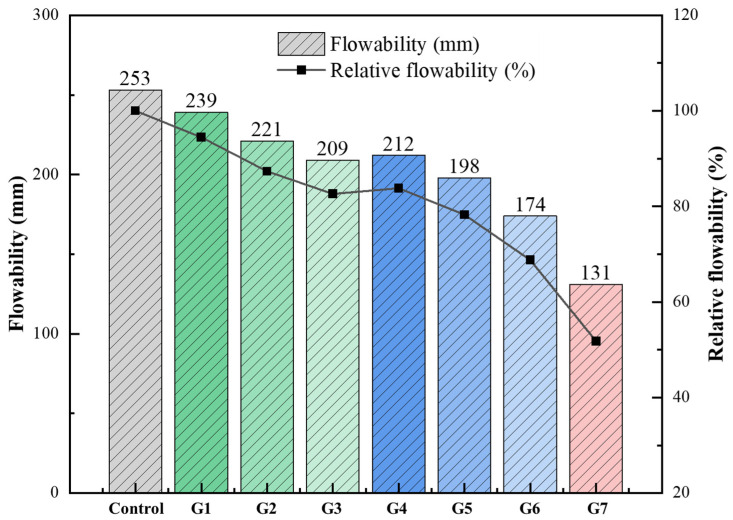
Effects of ISW and RPF on flowability of e-UHPC.

Figure 6 illustrates the effects of ISW and RPF incorporation on the flowability of e-UHPC. With increasing ISW and RPF contents, the flowability of e-UHPC gradually decreases. The influence of ISW on flowability is relatively limited, whereas RPF significantly affects flowability. When both ISW content and RPF substitution rate reach 50%, the flowability of e-UHPC decreases by more than 50%. In contrast, when only 50% ISW is added without RPF, the flowability decreases by only 17.30%, indicating that RPF content is the dominant factor affecting flowability.

The incorporation of ISW mainly increases the specific surface area of particles. Carbide slag and other materials consume a certain amount of free water during dissolution, leading to reduced mortar fluidity. The influence of RPF on flowability can be attributed to two aspects. On one hand, RPF has a larger specific surface area than steel fiber, and its strong adsorption capacity for the cement paste reduces the amount of free water. Therefore, excessive RPF incorporation greatly reduces mortar fluidity. Mohammad et al. [54] reported that incorporating 1.5% RPF by volume led to a 26.9% decrease in fluidity. On the other hand, the smooth surface of steel fibers contrasts with the rough surface of RPF, which increases frictional resistance between fibers and particles, thereby further decreasing flowability [55]. To ensure sufficient fluidity of e-UHPC for vibration compaction during construction, the RPF content should be limited to 30%.

### 3.2. Flexural Performance

**Figure 7:**
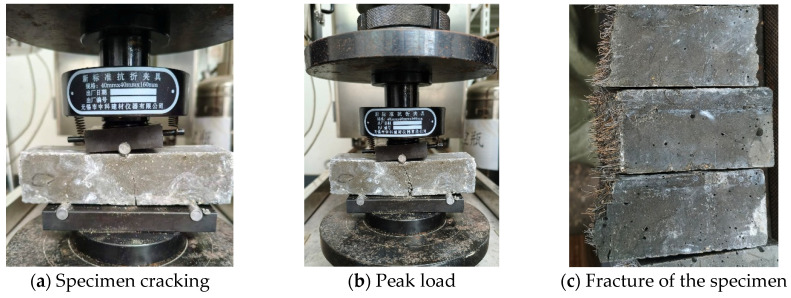
The flexural failure process of mortar specimens.

Due to fiber bridging, e-UHPC does not undergo sudden fracture during the flexural test. Instead, cracks first appear on the specimen surface (Figure 7a), then propagate to the full section height (Figure 7b). During crack propagation, fiber pull-out sounds can be heard, and eventually the specimen completely breaks apart (Figure 7c).

**Figure 8:**
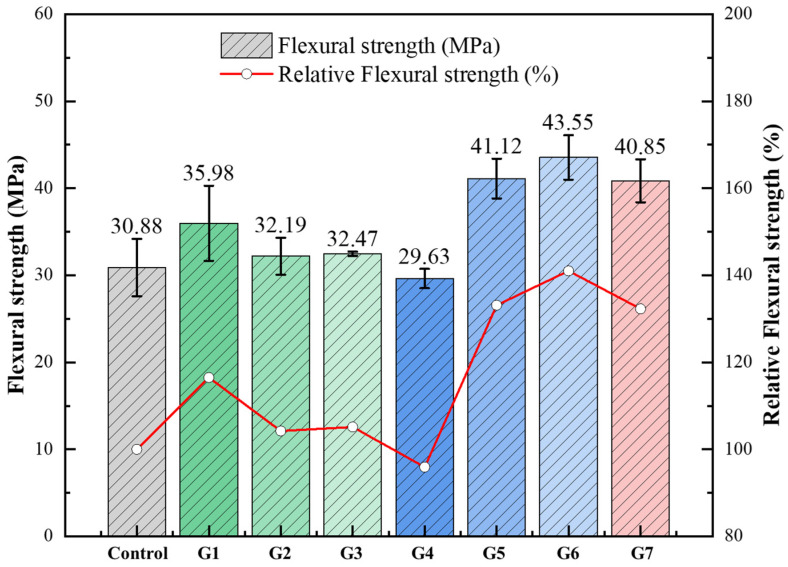
Effects of ISW and RPF on flexural strength of e-UHPC.

The flexural strength of e-UHPC cement mortar is shown in Figure 8 and Table 4.

The 7d flexural strength showed a non-monotonic numerical response to ISW replacement. The mean value increased from 30.88 ± 3.30 MPa for Control to 35.98 ± 4.31 MPa for G1 (10% ISW; +16.49%), and then decreased to 32.19 ± 2.12 MPa for G2 (30% ISW; +4.23%) and 32.47 ± 0.24 MPa for G3 (50% ISW; +5.14%). The flexural strength of cement-based materials depends on the density of the matrix itself and the bond between the paste and steel fibers. A large amount of C-S-H and C-A-S-H gels are formed through the reaction of carbide slag with slag, silica fume, and fly ash. These gels fill the internal pores of the matrix and densify the micro-interface structure of UHPC. Moreover, these hydration gels strengthen the interfacial transition zone between steel fibers and the paste, improving the bonding ability. Therefore, an appropriate amount of ISW can enhance the flexural strength of e-UHPC, which is consistent with previous studies [56]. In the range of 10–30%, the filler effect and pozzolanic reaction of ISW help densify the paste, improve fiber–matrix interface bonding, and control crack propagation, thereby enhancing material toughness. Among these, the 10% ISW content yields the most significant increase (approximately 16.5%), indicating that an appropriate admixture content promotes secondary hydration reactions, improves matrix compactness, and enhances fiber interface bonding [57].

However, due to the limited activation effect of carbide slag as an activator, excessive ISW incorporation reduces the content of cement hydration products and lowers the degree of matrix hydration. Additionally, the activation by carbide slag leads to rapid generation of hydration products that encapsulate unhydrated particles, hindering subsequent hydration. Consequently, the flexural strength of specimens with 30% and 50% ISW content is lower than that of specimens with 10% ISW content [48,58,59].

Within the 30% ISW matrix, replacing steel fiber with RPF also produced a non-monotonic numerical response. Relative to G2 (32.19 ± 2.12 MPa), G4 decreased to 29.63 ± 4.11 MPa (−7.96%), whereas G5 and G6 increased to 41.12 ± 6.28 MPa (+27.73%) and 43.55 ± 7.55 MPa (+35.29%), respectively. Previous studies have shown that multi-fiber synergistic effects can better improve the flexural strength of UHPC [60]. At a low RPF substitution rate (10%), RPF cannot form an effective hybrid network with steel fibers, and the elastic modulus and tensile strength of RPF are lower than those of steel fibers, thus weakening the flexural strength of e-UHPC. At this stage, RPF mainly contributes to crack control and has little contribution to strength [61]. When the RPF substitution rate reaches 30% or higher, the hybrid fiber system formed by RPF and steel fibers significantly enhances crack-bridging ability and improves flexural strength and toughness [62].

In summary, the introduction of ISW and RPF leads to distinct variations in flexural strength. An appropriate amount of mineral admixture optimizes particle gradation and improves matrix compactness through micro-filling and pozzolanic reactions, thereby enhancing fiber–matrix interface performance. When the ISW substitution rate is further increased to 30% and 50%, the flexural strength slightly decreases but remains higher than that of the control group; the dilution effect caused by the high substitution rate reduces the amount of hydration products. The higher mean flexural strengths at the 30% and 50% RPF replacement levels are consistent with, but do not demonstrate, a possible contribution from distributed crack bridging by the combined fiber system. The overall results indicate that appropriate mineral substitution rates and fiber mixing ratios have a synergistic optimization effect on the flexural performance of UHPC.

### 3.3. Compressive Performance

**Figure 9:**
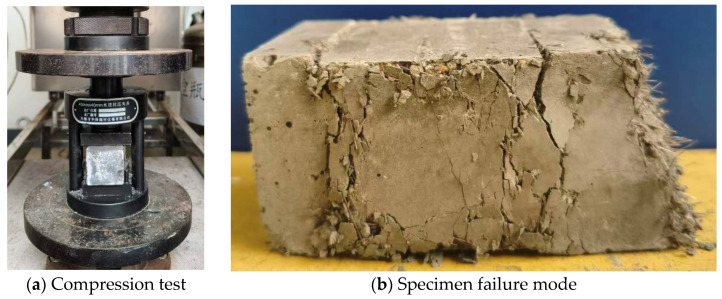
The compressive failure process of mortar specimens.

**Figure 10:**
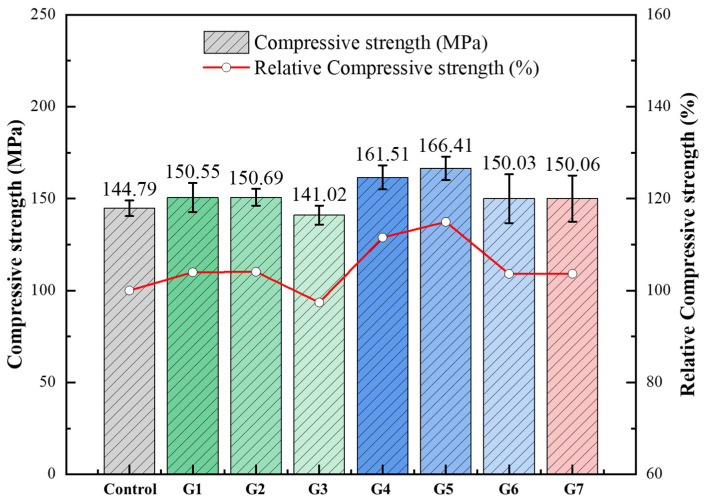
Effects of ISW and RPF on compressive strength of e-UHPC.

During compression, the failure mode and process of e-UHPC are similar to those of ordinary concrete, as shown in Figure 9b. The compressive strength results are presented in Figure 10 and Table 5.

The 7d compressive strength differed across the ISW series (*p* = 0.0326). Compared with Control (144.79 ± 4.31 MPa), the mean strengths of G1 and G2 were numerically higher at 150.55 ± 7.99 MPa (+3.98%) and 150.69 ± 4.59 MPa (+4.08%), whereas G3 decreased to 141.02 ± 5.15 MPa (−2.60%).

As seen in Figure 10, the effect of ISW content on compressive strength is similar to that on flexural strength. However, 30% ISW content leads to a greater increase in compressive strength, while 50% ISW content has a negative effect. ISW particles can fill the pores of UHPC, improving structural compactness and increasing compressive strength. Previous studies have shown that 20–30% slag incorporation promotes pozzolanic reactions, increases the formation of C-S-H gel, and optimizes microstructure and compactness [63,64,65]. Nevertheless, excessive ISW content severely dilutes the cement content, resulting in a lower degree of hydration [66]. The strength reduction at high ISW replacement levels was mainly attributed to cement dilution, incomplete ISW reaction, residual Ca(OH)_2_ accumulation, and the formation of a less compact matrix, rather than the formation of C-A-S-H itself [67].

RPF replacement had a statistically supported effect on compressive strength within the 30% ISW matrix (*p* = 0.0034). Relative to G2 (150.69 ± 4.59 MPa), G4 increased to 161.51 ± 6.50 MPa, a mean difference of 10.82 MPa (+7.18%). G5 reached the maximum value of 166.41 ± 6.37 MPa, corresponding to a mean difference of 15.72 MPa (+10.43%). By contrast, G6 (150.03 ± 13.27 MPa) did not differ from G2 (−0.44%) and exhibited a higher CV (8.85%).

Low-to-moderate RPF replacement increases the number of fibers available to bridge distributed microcracks. The material must withstand higher stress to form penetrating cracks, resulting in a significant increase in compressive strength [68,69]. However, excessive RPF incorporation leads to fiber agglomeration, creating defects within the material [70]. The significant increase in strength of G4 and G5 supports the existence of a beneficial compositional range, which may be due to a multi-scale crack-bridging mechanism, but the fiber dispersion and orientation were not measured.

The flexural strength of G2 was lower than that of G1, while its compressive strength was higher. This phenomenon can be attributed to the increased dosage of CC, which accelerated the reaction rate of slag and thus improved the compressive strength. Nevertheless, an excessively fast reaction rate may induce interfacial defects between fibers and the matrix, resulting in a lower flexural strength for G2 compared with G1.

The micro-filling effect and secondary hydration reaction of ISW improve matrix properties and compressive strength. When RPF replaces a portion of steel fibers to form a hybrid fiber system, the compressive strength is significantly enhanced. The maximum compressive strength (166.41 MPa) is achieved at 30% RPF replacement. This improvement may be due to the multi-scale crack-bridging system formed by the hybrid fibers of steel fibers and RPF, which effectively inhibits crack propagation. When the replacement rate of RPF is too high, the overall load-bearing capacity decreases due to the reduced number of steel fibers, resulting in a decrease in compressive strength.

### 3.4. Hydration Kinetics

**Figure 11:**
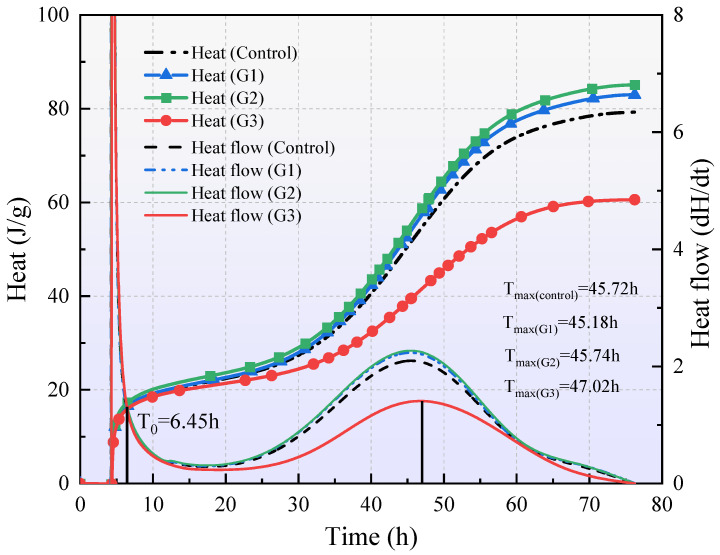
Effects of ISW on hydration heat of e-UHPC.

The hydration heat test reveals the hydration reaction process of different cementitious materials. Since RPF does not participate in cement hydration, only the standard groups, G1, G2 and G3, were tested to determine the effect of ISW on cement hydration. As shown in Figure 11, the shape of the hydration heat curve of UHPC remains almost unchanged upon adding different proportions of ISW. The similar shape of the cumulative heat release curves indicates that the dominant hydration reaction type does not change.

The main reactions of the silicate clinker phases can be represented by the following idealized equations:

(1) $$2(3CaO\cdot {SiO}_{2})+6H_{2}O\to 3CaO\cdot {2SiO}_{2}\cdot 3H_{2}O+3Ca{\left(OH\right)}_{2}$$

(2) $$2(2CaO\cdot {SiO}_{2})+4H_{2}O\to 3CaO\cdot {2SiO}_{2}\cdot 3H_{2}O+Ca{\left(OH\right)}_{2}$$

The hydration of C_3_S contributes strongly to the main early-age heat-flow peak, whereas C_2_S reacts more slowly and generates less Ca(OH)_2_.

After ISW addition, clinker hydration remained the dominant early-age reaction, while the latent hydraulic reaction of GGBS was promoted by the alkaline Ca(OH)_2_ supplied by CC, which is consistent with previous findings [71]. The resulting increase in alkalinity promotes the hydrolysis of the aluminosilicate glass network of GGBS. At the molecular scale, the hydroxyl-assisted cleavage of the bridging bonds can be represented schematically as:

(3) $$\equiv Si-O-Si\equiv +{OH}^{-}\to \equiv Si-OH+\equiv Si-O^{-}$$

(4) $$\equiv Si-O-Al\equiv +{OH}^{-}\to \equiv Si-OH+\equiv Al-O^{-}$$

(5) $$SiO_{2}+xCa{\left(OH\right)}_{2}+(n-x)H_{2}O\to xCaO\cdot {SiO}_{2}\cdot nH_{2}O$$

Meanwhile, dissolved aluminate species can substitute for some of the silicate tetrahedra in the gel structure, producing Al-containing C-(A)-S-H with a generally lower Ca/Si ratio than clinker-derived C-S-H. Mg released from GGBS may also participate in Mg-Al-containing secondary phases.

The calorimetry results were used to compare the low and high ISW replacement levels. The 10% ISW mixture showed a slight acceleration effect, whereas the 50% ISW mixture exhibited a clear dilution/inhibition effect. The G2 group shows the highest total heat release, indicating a higher degree of hydration; consequently, its flexural and compressive strengths are higher than those of the control group. This is because the addition of ISW provides an additional alkaline source, increasing the overall reaction rate and producing additional C-S-H gel, leading to higher strength [72,73,74]. The cumulative heat release of G3 was significantly lower than that of other test groups, resulting in a lower flexural strength compared to G1 and G2, and even a lower compressive strength than the control group. When ISW is used to replace cement at a 50% ratio, it can be observed that the exothermic rate slows down significantly, and the maximum exothermic time increases. The hydrated gel formed by the rapid reaction of slag hinders the diffusion of ions, ultimately leading to a decrease in the mechanical properties of the matrix, with a particularly significant decrease in compressive strength [75]. However, the dense gel structure produced by alkali-activated slag may have better bonding performance for fibers, resulting in a slight increase in the flexural strength of G3 compared to the cement control group.

However, when 10% and 30% of ISW are used to replace cement, due to the low content of ISW, cement hydration still plays a dominant role. The gel produced by alkali activation of slag serves as a reinforcement, enhancing the compressive strength of the matrix. Although the total heat release of G2 is the highest, the time to reach the maximum heat release rate (T_max_) has shifted to the right. This indicates that the incorporation of 30% ISW has significantly affected the diffusion stage of hydration, which explains why G2 has the highest compressive strength but lower flexural strength compared to G1. The rapid reaction of slag may result in the presence of a distinct transition zone within the matrix, and flexural strength is more sensitive to the homogeneity of the matrix’s strength.

The ISW content exhibits a dual effect on the hydration kinetics of e-UHPC, transitioning from an accelerator to an inhibitor. At a 30% replacement level, the Ca(OH)_2_ within the ISW activates the cement clinker and alkali-reactive powder. This promotes secondary hydration, leading to a greater yield of C-S-H gel and an increase in cumulative hydration heat. However, as the ISW dosage rises further, the hydration reaction becomes progressively suppressed due to the dilution of cement. This inhibition manifests as a slower hydration rate, a delayed heat release peak, and a reduction in hydration products, which ultimately compromises the mechanical strength.

### 3.5. Microstructural Evolution

**Figure 12:**
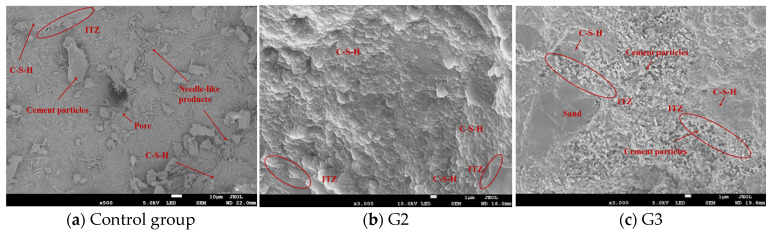
Microstructure of e-UHPC.

SEM was used to observe the morphology and interfacial transition zone of the paste under different mix proportions to explain the effect of ISW on matrix hydration. Figure 12a shows the paste morphology of the reference group. A small number of unhydrated particles are present, and the interfacial transition zone between the paste and aggregate is not obvious. Needle-like products can be seen, and the paste is dense without obvious defects. Figure 12b shows the microstructure of the G2 group. The products are mainly granular C-S-H gel, with almost no needle-like products. Particle stacking is extremely dense, with few pores or cracks. The paste exhibits a high degree of hydration, and no obvious unhydrated particles are observed. Comparing the reference group and G2 group, the G2 group shows a higher hydration degree, fewer defects and cracks, and denser stacking of hydration particles, accounting for its higher strength. This may be due to the filling of defects by an appropriate amount of ISW and the acceleration of cement hydration by Ca(OH)_2_ from carbide slag [63].

Figure 12c shows the paste morphology of the G3 group. A large number of unhydrated particles are present, and unhydrated cement particles are loosely distributed between quartz sand and C-S-H, forming an interfacial transition zone with many defects. Under external load, these interfacial transition zones, with high porosity and multiple defects, become weak links, resulting in decreased flexural and compressive strength. Excessive ISW dilutes the cement hydration products, and the activity of ISW itself is lower than that of cement, leading to a loose microstructure and reduced mechanical properties [76].

**Figure 13:**
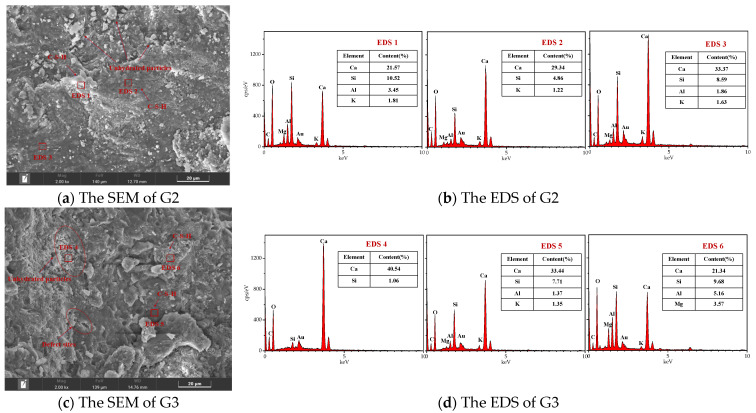
SEM-EDS of e-UHPC.

Figure 13 presents the SEM-EDS results for the G2 and G3 mixtures. G2 exhibits a relatively dense microstructure containing two morphologically different product regions. Compact and blocky regions are represented by EDS2 and EDS3, whereas EDS1 corresponds to a more granular region. In contrast, Figure 13c shows pronounced agglomeration of incompletely reacted particles in G3. These agglomerates produce heterogeneous and defective interfacial regions, which may contribute to the lower compressive strength of G3 compared with G2. EDS only counted elements with a content greater than 1%.

In G2, EDS1 has a Ca/Si ratio of 2.05, together with Al/Si and Mg/Si ratios of 0.33 and 0.17, respectively. The simultaneous presence of Ca, Si, Al, and Mg is consistent with an Al-containing C-(A)-S-H type hydrated gel formed during GGBS activation. By comparison, EDS2 and EDS3 exhibit substantially higher Ca/Si ratios of 6.73 and 8.58, respectively. This shows that these areas contain calcium-rich hydration products, which are related to the C-S-H gel formed during cement clinker hydration.

In G3, EDS4 exhibits an extremely high Ca/Si ratio of 38.25 and is therefore classified as a strongly Ca-rich region. Together with the increased portlandite reflections and refined portlandite content obtained from XRD, this region is associated with local Ca(OH)_2_ accumulation. EDS5 also has a relatively high Ca/Si ratio of 4.64, indicating a mixed Ca-rich gel/portlandite region. In contrast, EDS6 has a relatively lower Ca/Si ratio of 2.22 and substantially higher Al/Si and Mg/Si ratios of 0.54 and 0.37, respectively. Its composition is consistent with a GGBS-associated C-(A)-S-H-type reaction region containing possible Mg-bearing secondary products. These results indicate that GGBS activation still occurs in G3, but the reaction products are heterogeneously distributed and coexist with Ca-rich deposits and agglomerated incompletely reacted particles.

At an appropriate ISW replacement level, the filler effect of ISW and the formation of additional slag-associated reaction products contribute to pore filling and matrix densification. At the high replacement level, however, the heterogeneous coexistence of Ca-rich deposits and incompletely reacted particle agglomerates creates defective interfacial regions and adversely affects compressive strength.

### 3.6. Phase Composition by XRD

**Figure 14:**
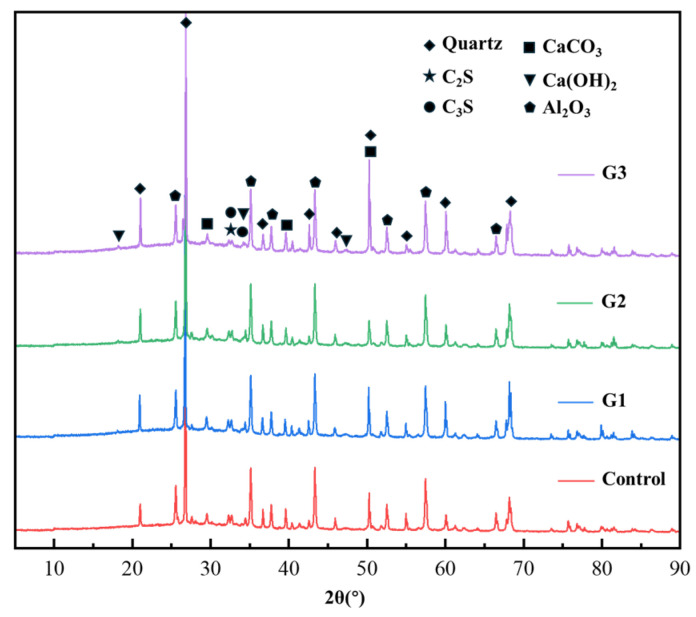
XRD of e-UHPC.

XRD was used to qualitatively analyze the hydration products of each specimen group [77]. Figure 14 presents the XRD patterns of e-UHPC under different ISW contents. The main phases identified are quartz (SiO_2_), calcium carbonate (CaCO_3_), calcium hydroxide (Ca(OH)_2_), alite (C_3_S), and belite (C_2_S). C-S-H gel is amorphous and typically produces a broad peak in the 2θ = 29°–32° region [78]. Therefore, Rietveld refinement was performed on the XRD patterns of the four groups of samples, and the relative content of each component is presented in Table 6.

Following ISW incorporation, the portlandite reflections became progressively more pronounced, which is consistent with the increasing contribution of Ca(OH)_2_ from CC. At low-to-moderate ISW replacement levels, Ca(OH)_2_ provides both OH^−^ and Ca^2+^, promoting the dissolution of the GGBS glass and the precipitation of additional C-(A)-S-H-type reaction products. At the highest replacement level, however, the amount of cement clinker is substantially reduced, while the supplied Ca(OH)_2_ cannot be completely consumed by GGBS. The increased portlandite content in G3 is consistent with the strongly Ca-rich EDS4 region, which is a local portlandite-rich deposit. The agglomerated incompletely reacted particles and defective interfaces observed by SEM further indicate that the reaction in G3 is spatially heterogeneous.

The diffraction intensities of residual alite(C_3_S) and belite (C_2_S) varied with ISW content, indicating that ISW incorporation modified the balance between clinker hydration and slag activation. It should be noted that the variation in C_3_S/C_2_S peak intensity cannot be interpreted solely as a direct change in clinker hydration degree, because it is also affected by cement dilution, slag reaction, and the relative proportion of hydration products. At a low ISW replacement level, Ca(OH)_2_ released from calcium carbide slag provides an alkaline environment that promotes the dissolution of GGBS and the formation of additional C-S-H/C-A-S-H gels. This agrees with the calorimetry results, where the G1 mixture exhibited a higher cumulative heat release than the control group. With increasing ISW content, however, the reduction in cement clinker becomes more pronounced, and excessive Ca(OH)_2_ cannot be fully consumed by the pozzolanic reaction. As a result, more unreacted particles and Ca(OH)_2_ precipitates remain in the matrix, weakening the compactness of the hydration products. Therefore, the improvement in strength at low-to-moderate ISW contents is mainly attributed to the combined effects of slag activation, additional amorphous gel formation, and matrix densification, whereas the strength reduction at high ISW content is governed by the dilution effect and incomplete reaction of ISW.

Regarding calcium carbonate content, the G1, G2, and G3 groups show a decreasing trend compared with the control group. CaCO_3_ mainly forms from carbonation of Ca(OH)_2_ during curing. For the G1 and G2 groups, the large consumption of Ca(OH)_2_ and the dense cementitious matrix formed by alkali-activated slag reduce CaCO_3_ content relative to the control group. For the G3 group, a large amount of Ca(OH)_2_ cannot react and precipitates; the hydration gel produced after the reaction encapsulates Ca(OH)_2_ particles, inhibiting contact with CO_2_, thus decreasing CaCO_3_ content while increasing Ca(OH)_2_ content.

The proportion of amorphous materials, in descending order, is G2 > G1 > control > G3, which is consistent with the trend of compressive strength. This indicates that amorphous materials represented by C-S-H and C-A-S-H gels are the main sources of compressive strength in e-UHPC. Due to the incorporation of reactive materials, more hydration gels (C-S-H and C-A-S-H) are generated in the G2 group, resulting in the highest strength.

XRD results indicate that appropriate ISW incorporation increases the formation of amorphous hydration products, mainly C-S-H and C-A-S-H gels, thereby improving matrix compactness and mechanical strength. However, excessive ISW incorporation leads to cement dilution and the accumulation of unreacted Ca(OH)_2_, which weakens the matrix and reduces strength.

### 3.7. Chemical Bonding Characteristics by FTIR

**Figure 15:**
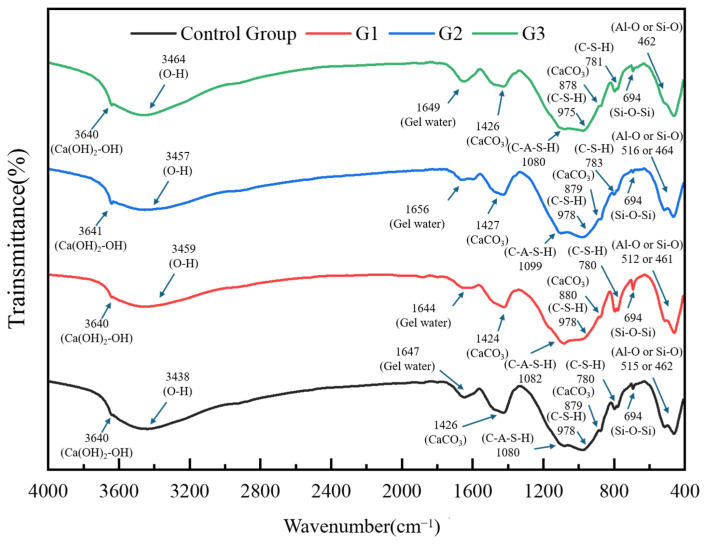
FTIR of e-UHPC.

FTIR provides information about molecular structure by measuring molecular vibration modes, allowing identification and characterization of internal functional groups. When infrared (IR) light passes through a sample, different functional groups absorb energy at specific frequencies corresponding to their vibration modes in the IR region of the electromagnetic spectrum [79]. This selective absorption leads to a decrease in transmitted IR intensity, reflecting the structural composition and quantity of hydration products [80,81]. Figure 15 presents the FTIR spectra of all e-UHPC groups over the full wavenumber range of approximately 400–4000 cm^−1^. All groups show obvious broad peaks in the 950 cm^−1^–1080 cm^−1^ region, which is one of the main peaks of cement hydration products. The region 980 cm^−1^–1000 cm^−1^ is mainly attributed to C-S-H gel, while 1000 cm^−1^–1080 cm^−1^ corresponds to C-A-S-H gel.

The characteristic region of C-A-S-H for the G1 group shifts from 1080 cm^−1^ to 1082 cm^−1^, and for the G2 group from 1080 cm^−1^ to 1099 cm^−1^. G1 (10% ISW) exhibits a higher peak in the 1099 cm^−1^ region, while the other groups show higher peaks in the 978 cm^−1^ region. The shift in the absorption peak toward a higher wavenumber indicates that dissolved aluminum participates in the hydration reaction and forms chain-structured C-A-S-H gel. The chain-like C-A-S-H gel possesses superior slip energy dissipation capacity, which endows the specimen in this group with higher flexural strength. Nevertheless, G2 and G3 with higher ISW dosages did not show this feature. Combined with the XRD results, it can be inferred that excessive ISW incorporation resulted in a highly alkaline environment and the accumulation of residual Ca(OH)_2_. This may have limited the further dissolution of aluminosilicate phases and hindered the continuous formation of C-A-S-H gel.

In the high-wavenumber region, broad absorption bands centered at approximately 3439–3457 cm^−1^ were observed in all groups. These bands are assigned to the O-H stretching vibration of hydrogen-bonded water associated with the hydration products. The corresponding H-O-H bending bands were observed at approximately 1644–1653 cm^−1^, demonstrating the presence of molecular water in all specimens. The slight variation in the position and shape of these bands suggests differences in the local hydrogen-bonding environments of water. A relatively sharp band was also observed at approximately 3640–3643 cm^−1^ and is attributed to the O-H stretching vibration of portlandite. This band was more pronounced in the G2 and G3 spectra than in the control and G1 spectra, qualitatively agreeing with the XRD results indicating greater residual Ca(OH)_2_ at relatively high ISW contents.

The main characteristic peak of calcium carbonate is observed in the 880 cm^−1^ region. The absorption peaks of the G1, G2, and G3 groups show a decreasing trend compared with the control group, consistent with XRD analysis. As ISW content increases, CaCO_3_ formation decreases while Ca(OH)_2_ content increases.

Overall, the FTIR results, together with the XRD analysis, indicate that an appropriate ISW dosage promoted the formation of C-S-H/C-A-S-H gels by providing a suitable alkaline environment for GGBS activation. This contributed to matrix densification and strength improvement. However, excessive ISW incorporation caused cement dilution and residual Ca(OH)_2_ accumulation, leaving more unreacted particles and internal pores in the matrix, which reduced compactness and mechanical strength.

### 3.8. Environmental and Economic Assessment

Cement and steel fibers make substantial contributions to the embodied carbon of conventional UHPC. Accordingly, this study conducted a simplified raw-material embodied-carbon assessment for 1 m^3^ of e-UHPC. The boundary included the production of cement, SF, FA, GGBS, CC, steel fibers, and RPF. Transportation, mixing, 90 °C steam curing, equipment, labor, construction, service life, and end-of-life stages were excluded. Mixing and steam-curing procedures were identical for all laboratory mixtures, so their omission isolates the effect of composition on the comparative results. Following the standard practice adopted by most researchers for carbon emission accounting, the total carbon emissions are calculated using a designated formula (6).

(6) $${\mathrm{CO}}_{2,\mathrm{total}}=\sum m_{i}\;\times \;EF_{i}$$

where *m_i_* is the material usage (kg/m^3^), $EF_{i}$ is the emission factor, and ${\mathrm{CO}}_{2,\mathrm{total}}$ is the total carbon emission ($\mathrm{kg}\;{\mathrm{CO}}_{2}\text{-}\mathrm{eq}/m^{3}$).

The carbon emission factors adopted in this study were derived from the Inventory of Carbon and Energy (ICE) database [82], as shown in Table 7. Since reliable emission factor data for CC were unavailable, relevant research reported by Horpibulsuk et al. [83] was referenced. SP, water, and sand are used in equal proportions, with a total cost of 151.94 CNY/m^3^.

Considering that e-UHPC exhibits both high strength and high absolute carbon emissions, it is also necessary to calculate the carbon emission per unit strength by formula (7). A coupled evaluation of environmental and mechanical indicators of the materials is required to select the optimal mix proportion of e-UHPC through comprehensive analysis.

(7) $${\mathrm{CO}}_{2,\mathrm{intensity}}=\frac{{\mathrm{CO}}_{2,\mathrm{total}}}{f_{c}}$$

where $\;{\mathrm{CO}}_{2,\mathrm{total}}$ is the total carbon emission ($\mathrm{kg}\;{\mathrm{CO}}_{2}\text{-}\mathrm{eq}/m^{3}$), *f*_c_ is the compressive strength of UHPC (MPa), and ${\mathrm{CO}}_{2,\mathrm{intensity}}$ is the carbon emission per unit strength, with the unit ($\mathrm{kg}\;{\mathrm{CO}}_{2}\text{-}\mathrm{eq}/\mathrm{MPa})$.

Costs are calculated based on the average purchase price in the building materials market in Eastern China. Because material prices, procurement scale, supplier, transport distance, and location vary substantially, the absolute values are not presented as universally transferable raw-material costs. As shown in Table 8, the total carbon emissions of each component in e-UHPC vary, with Control exhibiting the highest carbon emission of 852.29, and G7 presenting the lowest value of 453.34. The two most influential factors affecting the carbon emissions of each component are cement and steel fibers. As cement is progressively replaced by ISW, the reduction in cement content leads to a gradual decrease in carbon emissions. Meanwhile, as steel fibers are increasingly replaced by RPF, the reduction in steel fiber content further contributes to the decrease in carbon emissions. Consequently, the final carbon emission of e-UHPC is reduced by as much as 46.8% (G7), corresponding to approximately 398.95. Considering further the carbon emission per unit strength, it is observed that both the substitution of cement with ISW and the replacement of steel fibers with RPF lead to a reduction in the carbon emission per unit strength for all components, thereby lowering carbon emissions while maintaining compressive strength.

A comparison of the raw-material costs of each e-UHPC component reveals that as the replacement levels of cement and steel fibers increase, the cost gradually decreases. At the maximum substitution levels for both materials, the cost reduction is most pronounced, with G7 achieving a cost reduction of approximately 24.71%. This indicates that the use of ISW as a cement substitute and RPF as a replacement for steel fibers can yield favorable economic benefits in the production of e-UHPC.

Considering mechanical performance, flowability, carbon emissions, and raw-material cost, the most suitable mixture can be identified according to the intended application scenario. The mixture containing 30% ISW and 50% RPF achieved the highest flexural strength and is therefore more suitable for applications oriented towards flexural performance. The mixture containing 30% ISW and 30% RPF achieved the highest compressive strength while maintaining acceptable flowability, making it a balanced formulation for potential structural and underground mine reinforcement applications requiring high strength and construction reliability. In contrast, the mixture containing 50% ISW and 50% RPF achieved the greatest carbon emission and cost reductions while maintaining a compressive strength of approximately 150 MPa, making it more suitable for low-carbon and cost-sensitive applications.

## 4. Conclusions

This study systematically investigated the effects of industrial solid waste (ISW) and recycled plastic fiber (RPF) on the flowability, mechanical properties, hydration behavior, microstructure, carbon emissions, and raw-material cost of eco-friendly ultra-high-performance concrete (e-UHPC). Based on the experimental results and microstructural analyses, the following conclusions can be drawn:

1. The incorporation of ISW had a limited effect on the flowability of e-UHPC, whereas RPF significantly reduced flowability due to its higher water absorption capacity and increased interparticle friction. From the perspective of practical flowability, the RPF replacement ratio is recommended to be limited to 30%, although higher RPF contents may further improve flexural strength under specific mixture conditions.
2. ISW exhibited a clear dosage-dependent effect on the mechanical properties of e-UHPC. An appropriate ISW dosage promoted secondary hydration and improved matrix compactness, thereby contributing to strength development. However, excessive ISW replacement induced a dilution effect, which weakened the mechanical performance of e-UHPC.
3. Hydration heat analysis showed that 10% ISW accelerated early hydration and increased cumulative heat release, whereas 50% ISW delayed the hydration process and reduced total heat release. This indicates that the dominant role of ISW gradually changes from hydration promotion to dilution at high replacement levels.
4. SEM, XRD, and FTIR analyses confirmed that an appropriate ISW dosage promoted the formation of C-S-H/C-A-S-H gels and refined the microstructure of e-UHPC. In contrast, excessive ISW incorporation resulted in more unreacted particles and a looser interfacial transition zone, thereby reducing matrix compactness and mechanical strength.
5. While hybrid steel fibers and RPF enhanced e-UHPC’s mechanical performance, the most suitable mixture depends on application needs. The 30% ISW and 50% RPF mix maximized flexural strength, while 30% ISW and 30% RPF offered the best strength-flowability balance for structural use. Conversely, 50% ISW and 50% RPF provided the greatest cost and carbon reductions, suiting low-carbon, cost-sensitive applications.

The study considered only 10%, 30%, and 50% cement replacement at a fixed 5:1 GGBS/CC ratio and 90 °C steam curing, limiting optimization and extrapolation to ambient-cured or cast-in-place UHPC. Mine-relevant durability (sulfate resistance, chloride penetration, shrinkage, and freeze–thaw) was untested, leaving long-term suitability unverified. Characterization covered only compressive/flexural strength and flow spread, excluding tensile strength, elastic modulus, load–displacement response, fracture energy, and rheology (yield stress, plastic viscosity, and thixotropy). These gaps require investigation under service-relevant conditions before engineering use.

## Figures and Tables

**Table 1:** Technical specifications for cement.

Surface Area (m^2^/kg)	Initial Setting Time (min)	Setting Time (min)	Flexural Strength (MPa)		Compressive Strength (MPa)	
			3d	28d	3d	28d
387	186	287	6.7	9.5	31.5	68.4

**Table 2:** XRF test results (wt.%).

Component	Na_2_O	MgO	Al_2_O_3_	SiO_2_	CaO	Fe_2_O_3_	Loss
SF	0.119	0.057	0.131	98.712	0.352	0.097	0.015
FA	0.718	1.351	31.364	48.369	7.147	0.045	3.441
GGBS	0.971	12.356	14.669	39.767	28.974	1.136	0.943
CC	0.172	0.027	0.274	1.495	71.138	0.021	21.749

**Table 3:** Mix proportion and fresh properties of specimens (1 m^3^).

ID	Cement (kg)	FA (kg)	SF (kg)	ISW (kg)	SP (kg)	Steel Fiber (kg)	RPF (kg)	Water (kg)	Sand (kg)
Control	709	248	71	0	11.6	109	0	162	928
G1	638.1	248	71	70.9	11.6	109	0	162	928
G2	496.3	248	71	212.7	11.6	109	0	162	928
G3	354.5	248	71	354.5	11.6	109	0	162	928
G4	496.3	248	71	212.7	11.6	98.1	1.28	162	928
G5	496.3	248	71	212.7	11.6	76.3	3.83	162	928
G6	496.3	248	71	212.7	11.6	54.5	6.39	162	928
G7	354.5	248	71	354.5	11.6	54.5	6.39	162	928

**Table 4:** Flexural strength statistics.

Specimen	ISW/RPF(%)	Flexural Strength (MPa)	Standard Deviation (SD)	Coefficient of Variation (CV,%)	Standard Error (SE)	Welch Test-*p* Value
Control	0/0	30.88	3.31	10.67	1.90	0.628
G1	10/0	35.98	4.31	11.99	2.49	
G2	30/0	32.19	2.12	6.6	1.23	
G3	50/0	32.47	0.24	0.74	0.14	
G4	30/10	29.63	4.11	13.89	2.38	0.1387
G5	30/30	41.12	6.28	15.28	3.63	
G6	30/50	43.55	7.55	17.34	4.36	
G7	50/50	40.85	2.46	6.03	1.42	

**Table 5:** Compressive strength statistics.

Specimen	ISW/RPF(%)	Compressive Strength (MPa)	Standard Deviation (SD)	Coefficient of Variation (CV,%)	Standard Error (SE)	Welch Test-*p* Value
Control	0/0	144.79	4.31	2.98	1.76	0.0326
G1	10/0	150.55	7.99	5.31	3.26	
G2	30/0	150.69	4.59	3.04	1.87	
G3	50/0	141.02	5.15	3.66	2.1	
G4	30/10	161.51	6.50	4.03	2.66	0.0034
G5	30/30	166.41	6.37	3.83	2.6	
G6	30/50	150.03	13.27	8.85	5.42	
G7	50/50	150.06	12.52	8.34	5.11	

**Table 6:** Relative content of each component (%).

ID	Quartz	Al_2_O_3_	CaCO_3_	Ca(OH)_2_	C_2_S	C_3_S	Amorphous (C-S-H)	GOF
Control	33.83	15.25	12.58	4.19	5.60	5.67	22.88	12.16
G1	34.12	15.42	11.75	4.24	5.63	5.69	23.15	11.59
G2	34.29	15.30	11.27	4.55	5.11	4.81	24.67	11.34
G3	38.71	17.01	11.08	5.33	3.55	3.24	21.08	10.65

**Table 7:** Carbon emission factors and material cost of materials in e-UHPC.

Materials	Cement	FA	SF	CC	GGBS	Steel Fiber	RPF
$EF_{i}$	0.9 [82]	0.02 [82]	0.03 [82]	0.007 [83]	0.07 [82]	1.9 [82]	0.4 [82]
Material cost (CNY/t)	325	260	700	320	276	4800	4000

**Table 8:** Comparison of carbon emissions and costs of each component in e-UHPC.

ID	Total CO_2_ (kg/m^3^)	Cement Reduction (kg/m^3^)	Steel-Fiber Reduction (kg/m^3^)	Compressive Strength (MPa)	CO_2_ Intensity (kg CO_2_-eq/(MPa·m^3^)	Estimated Material Cost (CNY/m^3^)	Cost Efficiency (%)
Control	852.29	0	0	144.79	5.89	1019.75	0.00
G1	792.70	63.81	0	150.55	5.27	1016.56	0.31
G2	673.52	191.43	0	150.69	4.47	1010.17	0.94
G3	554.33	319.05	0	141.02	3.93	1003.79	1.56
G4	653.32	191.43	20.71	161.51	4.05	962.92	5.57
G5	612.92	191.43	62.13	166.41	3.68	868.53	14.83
G6	572.52	191.43	103.55	150.03	3.82	774.13	24.09
G7	453.34	319.05	103.55	150.06	3.02	767.75	24.71

## Data Availability

The original contributions presented in this study are included in the article. Further inquiries can be directed to the corresponding author.
